# Comprehensive Senior Technology Acceptance Model of Daily Living Assistive Technology for Older Adults With Frailty: Cross-sectional Study

**DOI:** 10.2196/41935

**Published:** 2023-04-10

**Authors:** Hye Ri Shin, Sa Rang Um, Hee Jeong Yoon, Eun Young Choi, Won Chul Shin, Hee Yun Lee, Young Sun Kim

**Affiliations:** 1 Department of Gerontology, AgeTech-Service Convergence Major Kyung Hee University Yongin Republic of Korea; 2 School of Global Public Health New York University New York, NY United States; 3 Department of Neurology Kyung Hee University Hospital at Gangdong Seoul Republic of Korea; 4 School of Social Work University of Alabama Tuscaloosa, AL United States

**Keywords:** senior technology acceptance model, daily living assistive technologies, frailty, older adults

## Abstract

**Background:**

There are considerable gaps between the need for assistive technologies and the actual adoption of these technologies among older adults, although older adults are among the groups that most need assistive technologies. Consequently, research is needed in this area because older adults’ technology acceptance and influencing factors may differ depending on their level of frailty.

**Objective:**

The objective of this study was to compare frail, prefrail, and robust groups of South Korean adults regarding their behavioral intention to use daily living assistive technologies and the affecting factors—namely, technological context factors, health contexts and abilities, and attitudinal factors—based on a comprehensive senior technology acceptance model.

**Methods:**

A nationwide sample of 500 older South Korean adults (aged 55-92 years) was analyzed, and multivariate linear regression analyses of the robust, prefrail, and frail groups were performed. The independent and dependent variables consisted of 3 factors based on previous studies. First, technological context factors consisted of gerontechnology self-efficacy, gerontechnology anxiety, and facilitating conditions. Second, health contexts and abilities consisted of self-reported health conditions, cognitive ability, social relationships, psychological function, and physical function. Third and last, attitudinal factors consisted of behavioral intention to use assistive technologies, attitude toward use, perceived usefulness (PU), and perceived ease of use (PEOU).

**Results:**

The results of the analyses showed that technological context factors such as gerontechnology self-efficacy, health contexts and abilities such as self-reported health conditions and psychological function, and attitudinal factors such as attitude toward use, PU, and PEOU had significant effects on behavioral intention to use daily living assistive technologies. In particular, gerontechnology self-efficacy had a significant relationship with behavioral intention to use these technologies in the robust (*r*=0.120; *P*=.03) and prefrail (*r*=0.331; *P*<.001) groups. Psychological function (life satisfaction) had a significant relationship with behavioral intention to use these technologies in the robust group (*r*=–0.040; *P*=.02). Self-reported health conditions had a significant relationship with behavioral intention to use these technologies in the prefrail group (*r*=–0.169; *P*=.01). Although each group had a different significant relationship with the variables, attitudinal factors such as attitude toward use affected all groups (robust group: *r*=0.190; *P*=.03; prefrail group: *r*=0.235; *P*=.006; and frail group: *r*=0.526; *P*=.002). In addition, PU and PEOU in the attitudinal factors had a significant relationship with behavioral intention to use assistive technologies in the robust (PU: *r*=0.160; *P*=.01; and PEOU: *r*=0.350; *P*<.001) and prefrail (PU: *r*=0.265; *P*<.001; and PEOU: *r*=0.120; *P*=.04) groups.

**Conclusions:**

This study found that the comprehensive senior technology acceptance model of daily living assistive technologies had different associations according to the frailty group. These findings provided insights into the consideration of interventions with daily living assistive technologies for older adults with varying levels of frailty.

## Introduction

### Background

Assistive technology is an umbrella term that refers to any tool, device, aid, or service that people can use to live independent and healthy lives by maintaining or improving the functioning needed for daily activities; this technology ranges from mobility and hearing aids to computer software and electrical devices [1]. According to representative population surveys from 29 countries, >2.5 billion people are estimated to benefit from ≥1 assistive technology products, and this number is expected to increase to >3.5 billion by 2050 with the growth of aging populations and increase in the prevalence of noncommunicable diseases [2]. Considering that aging is typically related to significant declines in physical and mental capacities, as well as a rising risk of developing diseases, older adults are among the groups that most need assistive technologies [3]. However, there are considerable gaps between the need for assistive technologies and the actual adoption of these technologies among older adults [4]. Without adequate access to assistive technology products, people in need are likely to be confined to their homes, which increases the risk of poverty and social isolation [5]. Thus, identifying and targeting the factors that can improve older adults’ access to assistive technologies are urgently necessary to satisfy the aforementioned unmet needs.

Previous research identified a series of individual-level barriers that are related to the adoption of assistive technologies: age, gender, a lack of awareness, socioeconomic status, and living environment [6,7]. However, although there is great heterogeneity in aging such that individuals increasingly differ in their patterns of health status and functioning with age [8], little is known about the different needs for, and adoption of, assistive technologies regarding the different stages of the aging process [9]. This calls for further research to examine the multidimensional predictors of assistive technology use in terms of one’s functional status.

Frailty is prevalent in old age and is defined as a complex state of increased vulnerability because of the adverse health outcomes associated with aging [10,11]. Many older people experience the loss of physical or mental health, which may require the use of assistive technologies such as assistive walkers, hearing aids, and electric beds [12]. Although older people may need assistive technologies because of poor health, they may also be reluctant to use them. The low acceptance of assistive technologies could lead older adults to limit or stop leaving the home and thus become homebound, worsening their health [13-15]. Assistive technologies are important for older adults with frailty, and several studies have investigated frailty and the use of technology.

Keränen et al [16] analyzed the differences in information and communication technologies use, attitudes, and reasons for nonuse among older adults categorized as physically frail, prefrail, and robust, and they found that older adults with frailty were less likely to use information and communication technologies than robust people. In addition, Buccoliero and Bellio [17] analyzed the factors that affect the adoption of technologies for health by older adults. Research showed that, in the case of health technologies, frailty negatively affects perceived ease of use (PEOU) and perceived usefulness (PU), PU positively affects behavioral intention to use health technologies, and PEOU negatively affects behavioral intention to use these technologies [17]. Interestingly, Lee et al [18] found that adults with frailty were less likely to use web-based health resources than healthy adults, but adults with frailty who used the internet alone or with assistance were more likely to obtain web-based health information and advice.

The technology acceptance model (TAM), which is one of the most widely used theoretical frameworks used to explain the factors affecting users’ adoption of new technologies [19-21], posits that various external variables influence the PU and PEOU of technologies, which, in turn, shape the users’ attitudes toward technology and their behavioral intention to use it, ultimately leading to actual use. Expanding the TAM, Chen and Chan [22] developed the senior TAM (STAM) to better identify a broad range of factors that are associated with older adults’ technology adoption. The authors conceptualized that the distinct characteristics of older adults, including physical, psychological, and social aspects related to aging, would affect older adults’ interactions with technology. Specifically, the STAM added the 8 factors described in Textbox 1 as the key predictors of PU and PEOU, as well as the behavioral intention to use technology.

**Key predictors of perceived usefulness and perceived ease of use, as well as the behavioral intention to use technology.**

Gerontechnology self-efficacy

- The extent to which older adults feel that they can use technology to improve their independent living and social engagement within the context of good health, comfort, and safety [23]. It was found to be significantly associated with the behavioral intention to use technology and ease of use [22,24].

Gerontechnology anxiety

- The anxiety that older adults feel about using technology [23], which leads to hesitation in the adoption of technology [25].

Facilitating conditions

- The belief that older adults have that there is an organizational and technological foundation to support their use of technology, with more facilitating conditions encouraging use [26].

Self-reported health conditions, physical functioning, and cognitive ability

- These are included because better health and functioning statuses are likely to have positive relationships with perceived usefulness and perceived ease of use of technology [22,23].

Social relationships and attitudes to life and satisfaction

- These are psychosocial factors that can increase the adoption of new technologies because social network members and more positive attitudes can encourage older adults to buy technological devices and facilitate their use [27,28].

### Objectives

According to the aforementioned studies, older adults’ technology acceptance and influencing factors may differ depending on the level of frailty, indicating that additional research is needed. In this study, we aimed to compare frail, prefrail, and robust groups regarding their behavioral intention to use daily living assistive technologies and the factors that affect it (technological context factors, health contexts and abilities, and attitudinal factors).

## Methods

### Data Source and Participants

This study aimed to examine the comprehensive factors that affect the behavioral intention of older adults in need of care to use daily living assistive technologies. Cross-sectional data were acquired as part of the technology adoption study of middle-aged and older South Koreans conducted by the department of gerontology at Kyung Hee University. The study is a nationwide, face-to-face survey of community-dwelling South Koreans aged ≥55 years that is conducted to understand the status of technology use and the acceptance of technology by older adults. The Hankook Research Company collected data on the web from September 16, 2019, to October 11, 2019, in 17 representative cities and provinces in South Korea using a stratified cluster random sampling technique. A total of 500 participants completed structured questionnaires on technology use, health status, psychosocial factors, and other sociodemographic characteristics.

Specifically, the participants were sampled using the cluster sampling method based on the national basic district of each city and province. Investigators, who completed professional training and education, conducted a 1:1 face-to-face interview by sequentially visiting nearby households, starting with the community center in the surveyed area. If absent, occupants were contacted up to 3 times.

The final analyzed sample had no missing information on the variables of interest for this study. The sample of 500 participants included 226 (45.2%) robust older adults, 212 (42.4%) older adults categorized as prefrail group, and 62 (12.4%) older adults with frailty. The participants were classified into robust, prefrail, and frail using the 5 categories (fatigue, resistance, ambulation, illnesses, and loss of weight) of the simple frailty questionnaire measurement method proposed by Morley et al [29].

### Ethics Approval, Informed Consent, and Participation

The study and all procedures were approved by the institutional review board of Kyung Hee University (KHGIRB-19-195). All participants consented to participate in the survey by telephone before participating in the survey. Written guidance was provided to them before the start of the survey, and consent was obtained again on the written informed consent form. In addition, the participants’ information was deidentified, and a sum of ₩3000 (US $2.44 in 2022) was offered to participants as monetary remuneration.

### Measures

There are various variables in the STAM. We used 3 categories (attitudinal factors, technological context factors, and health contexts and abilities) based on the studies by Chen and Chan [22] and Jarvis et al [30].

Most of the scales and items adopted for this survey have been widely used and validated in prior empirical studies. However, some items were modified to take the context of this research into account. The items for all variables except life satisfaction (LS) and physical function (activity of daily living) were measured using a 5-point Likert scale ranging from 1 (strongly disagree) to 5 (strongly agree). LS was measured using a 4-point Likert scale ranging from 1 (strongly disagree) to 4 (strongly agree), and instrumental activity of daily living (IADL) scores were assessed using a 3-point scale, based on the help required for each activity: 1=completely independent, 2=some help needed, and 3=completely dependent. The variables, items, their Cronbach α values, and sources are presented in Multimedia Appendix 1 [21,22,26,31-37]. Additionally, the mean, SE, and 95% CI of the variables are in Multimedia Appendix 2.

### Analytical Strategy

Before all analyses, we confirmed that the measured items had internal reliability and that they were mostly good in this sample (Cronbach α=.69-.90). First, descriptive statistics were calculated to review the demographic characteristics of the full sample. Next, we examined differences among the 3 groups using ANOVA. Finally, multivariate linear regression analyses were performed separately by group (robust, prefrail, and frail) to investigate the independent effects of technology acceptance regarding daily living assistive technologies. Groups of variables were entered in a series of steps: (1) demographic factors (age, gender, education, spouse, working status, and household income) that were previously reported to be related to the dependent variable; (2) attitudinal factors (attitude toward use [AT], PU, and PEOU); (3) technological context factors (gerontechnology self-efficacy [SE], gerontechnology anxiety [ANX], and facilitating conditions [FC]); and (4) health contexts and abilities (self-reported health conditions [HC], cognitive ability [CA], social relationships, attitude toward aging [ATT], LS, and IADL). In addition, a structural equation model was developed to verify the validity of the overall model. The results of hierarchical regression analysis and the structural equations are presented in Multimedia Appendices 3 and 4, respectively. All analyses were conducted using Stata (version 17.0; StataCorp LLC).

## Results

### Sample Characteristics

The descriptive characteristics of the study sample are summarized in Table 1. Of the 500 respondents, 269 (53.8%) were women. The average age of the respondents was 66.87 (SD 8.72) years. Of the 500 respondents, 215 (43%) lived in large cities. In recent years, the educational attainment of older adults in South Korea has risen, and 40% (200/500) of the respondents had graduated from high school. Three-quarters of the respondents (366/500, 73.2%) were married. Moreover, more than half of the respondents (319/500, 63.8%) were working, and the monthly household income of the total sample was approximately ₩2,923,800 (US $2376.49).

**Table 1:** Respondents’ demographic profile (N=500).

Characteristics		Values, n (%)		Values, mean (SD; range)			
**Gender**					1.54 (0.50; 1-2)		
	Men	231 (46.2)					
	Women	269 (53.8)					
Age (years)		N/A^a^		66.87 (8.72; 55-92)			
**Residence**					1.81 (0.80; 1-3)		
	Large city		215 (43)				
	Medium or small city		166 (33.2)				
	Rural		119 (23.8)				
**Educational attainment**					3.28 (1.09; 1-5)		
	No formal education	27 (5.4)					
	Elementary school	114 (22.8)					
	Middle school	106 (21.2)					
	High school	200 (40)					
	College or above	53 (10.6)					
**Marital status**					0.73 (0.44; 0-1)		
	Unmarried	134 (26.8)					
	Married	366 (73.2)					
**Employment status**					0.64 (0.48; 0-1)		
	Working	319 (63.8)					
	Not working	181 (36.2)					
Monthly household income^b^		N/A		292.38 (216.56; 0-2000)			

^a^N/A: not applicable.

^b^Unit: ₩10,000 (US $8.35 in 2022).

### The STAM Results According to the Frailty Group

#### Overview

Table 2 presents multiple comparisons across the 3 groups (robust, prefrail, and frail) based on the ANOVA results. Overall, the robust group had the highest score for attitudinal factors, technological context factors, and health contexts and abilities, except for ANX and physical function (IADL).

**Table 2:** The senior technology acceptance model results by group (N=500).

		Robust group (n=226), mean (SD)	Prefrail group (n=212), mean (SD)	Frail group (n=62), mean (SD)	*F* test (*df*)	*P* value
**Attitudinal factors**						
	Behavioral intention to use technology	7.27 (1.77)	6.94 (1.72)	6.24 (1.96)	8.39 (2)	<.001
	Attitude toward use	7.58 (1.55)	7.40 (1.47)	7.08 (1.49)	2.75 (2)	.07
	Perceived usefulness	11.51 (2.11)	11.27 (2.15)	11.02 (1.89)	1.60 (2)	.20
	Perceived ease of use	6.91 (1.80)	6.18 (1.79)	4.90 (2.04)	30.94 (2)	<.001
**Technological context factors**						
	Gerontechnology self-efficacy	7.53 (1.80)	6.93 (1.99)	5.53 (2.23)	26.16 (2)	<.001
	Gerontechnology anxiety	4.55 (1.99)	5.35 (1.85)	6.76 (2.09)	33.07 (2)	<.001
	Facilitating conditions	9.39 (2.87)	8.55 (2.80)	6.44 (2.50)	27.53 (2)	<.001
**Health contexts and abilities**						
	Self-reported health conditions	7.61 (1.23)	7.07 (1.36)	5.56 (1.47)	59.28 (2)	<.001
	Cognitive ability	19.50 (1.22)	19.33 (0.99)	17.98 (1.94)	36.81 (2)	<.001
	Social relationships	6.59 (2.41)	6.76 (2.29)	6.71 (2.34)	0.28 (2)	.76
	Psychological function 1 (attitude toward aging)	76.01 (7.06)	73.79 (6.78)	69.47 (7.51)	22.04 (2)	<.001
	Psychological function 2 (life satisfaction)	43.24 (5.42)	41.03 (6.17)	37.94 (5.77)	22.52 (2)	<.001
	Physical function (instrumental activity of daily living)	10.19 (0.89)	10.28 (0.99)	11.90 (3.16)	37.92 (2)	<.001

#### Attitudinal Factors

The robust group had the highest score for behavioral intention to use technology, AT, and PEOU (behavioral intention to use technology: mean 7.27, SD 1.77; AT: mean 7.58, SD 1.55; and PEOU: mean 6.91, SD 1.80). Moreover, the prefrail group had the second highest score in each of these same variables (behavioral intention to use technology: mean 6.94, SD 1.72; AT: mean 7.40, SD 1.47; and PEOU: mean 6.18, SD 1.79).

#### Technological Context Factors

The SE and FC scores were high in the following descending order: robust group (SE: mean 7.53, SD 1.80; and FC: mean 9.39, SD 2.87), prefrail group (SE: mean 6.93, SD 1.99; and FC: mean 8.55, SD 2.80), and frail group (SE: mean 5.53, SD 2.23; and FC: mean 6.44, SD 2.50). By contrast, the ANX scores were low in the following ascending order: robust group (mean 4.55, SD 1.99), prefrail group (mean 5.35, SD 1.85), and frail group (mean 6.76, SD 2.09).

#### Health Contexts and Abilities

HC, CA, psychological function 1 (ATT), and psychological function 2 (LS) scores had the same patterns as the SE and FC scores. In these variables, the robust group had the highest scores (HC: mean 7.61, SD 1.23; CA: mean 19.50, SD 1.22; ATT: mean 76.01, SD 7.06; and LS: mean 43.24, SD 5.42), and the frail group had the lowest scores (HC: mean 5.56, SD 1.47; CA: mean 17.98, SD 1.94; ATT: mean 69.47, SD 7.51; and LS: mean 37.94, SD 5.77). Older adults with physical limitations showed the opposite results, and the frail group had the highest physical function (IADL) score (mean 11.90, SD 3.16).

### Adapted STAM Testing

After adjusting for demographic factors, several STAM factors were found to be significantly associated with the behavioral intention to use daily living assistive technologies (Table 3). When the robust group (*r*=0.120; *P*=.03) and prefrail group (*r*=0.331; *P*<.001) had a higher SE score, they had a higher behavioral intention to use technology. With this result, we confirmed that, in healthier people, there was a significant relationship between SE and the behavioral intention to use daily living assistive technologies. From these results, we found that the robust group and the prefrail group had the characteristic of wanting to use daily living assistive technologies to seize the initiative after they had learned how to use the devices on their own.

Furthermore, HC scores were negatively associated with the behavioral intention to use daily living assistive technologies in the prefrail group (*r*=–0.169; *P*=.01), that is, we could interpret that the willingness to use technology to support daily life was more likely to increase in people whose health gradually became worse, increasing their need for care. This can also be seen from the result related to having a spouse (*r*=–0.715; *P*=.001). The group of older adults categorized as prefrail who did not have a spouse and had poor subjective health status had a higher need for care, and therefore it seemed that they had a higher intention to use a technological device.

**Table 3:** Predictions of behavioral intention to use assistive technologies by group (N=500).

		Robust group^a^ (n=226)						Prefrail group^b^ (n=212)							Frail group^c^ (n=62)								
		*r* (β)			*P* value			*r* (β)			*P* value			*r* (β)				*P* value					
**Demographic factors**																							
	Age			−0.020 (−.064)			.24			0.025 (.127)			.05				−0.007 (−.026)			.85			
	Gender			0.240 (.068)			.14			0.101 (.029)			.55				−0.165 (−.040)			.74			
	Education			−0.010 (.004)			.94			0.132 (.082)			.18				0.129 (.055)			.67			
	Spouse			−0.220 (−.049)			.28			−0.715 (−.186)			.001				−0.074 (−.019)			.87			
	Working status			−0.230 (−.057)			.22			0.066 (.018)			.75				0.421 (.091)			.46			
	Household income			0.000 (.035)			.48			0.000 (.071)			.18				−0.002 (−.169)			.17			
**Attitudinal factors**																							
	Attitude toward use	0.190 (.163)			.03			0.235 (.201)			.006			0.526 (.399)				.002					
	Perceived usefulness	0.160 (.165)			.01			0.265 (.276)			<.001			0.086 (.089)				.70					
	Perceived ease of use	0.350 (.415)			<.001			0.120 (.149)			.04			0.170 (.165)				.22					
**Technological context factors**																							
	Gerontechnology self-efficacy	0.120 (.124)			.03			0.331 (.382)			<.001			0.068 (.077)				.65					
	Gerontechnology anxiety	0.010 (.015)			.77			−0.028 (−.030)			.58			−0.059 (−.063)				.65					
	Facilitating conditions	0.050 (.073)			.24			−0.021 (.034)			.64			0.192 (.245)				.14					
**Health contexts and abilities**																							
	Self-reported health conditions		0.070 (.051)			.27			−0.169 (−.133)			.01				−0.044 (−.033)			.80				
	Cognitive ability		0.080 (.053)			.23			0.107 (.062)			.20				0.034 (.034)			.75				
	Social relationships		0.050 (.071)			.12			−0.020 (−.027)			.59				0.031 (.037)			.75				
	Psychological function 1 (attitude toward aging)		−0.010 (−.020)			.70			0.017 (.066)			.23				0.007 (.028)			.84				
	Psychological function 2 (life satisfaction)		−0.040 (−.123)			.02			−0.003 (−.010)			.86				0.007 (.021)			.89				
	Physical function (instrumental activity of daily living)		−0.020 (−.012)			.79			−0.085 (.049)			.31				0.058 (.093)			.52				

^a^*F*_18_=21.23 (*P*<.001), *R*^2^=0.650, adjusted *R*^2^=0.620.

^b^*F*_18_=18.79 (*P*<.001), *R*^2^=0.637, adjusted *R*^2^=0.603.

^c^*F*_18_=4.57 (*P*<.001), *R*^2^=0.657, adjusted *R*^2^=0.513.

In addition, PEOU, PU, and AT scores had significant relationships with the intention to use daily living assistive technologies in the robust group (PEOU: *r*=0.350; *P*<.001; PU: *r*=0.160; *P*=.01; and AT: *r*=0.190; *P*=.03) and the prefrail group (PEOU: *r*=0.120; *P*=.04; PU: *r*=0.265; *P*<.001; and AT: *r*=0.235; *P*=.006). In previous studies, the factors that influenced the intention to use various devices and technologies were also found to be the same for the intentions of robust older adults and older adults categorized as prefrail to use daily living assistive technologies. In particular, the influences of these factors on the intention to use technology were also mostly high, as we can see in the standardized β results (robust group: PEOU: β=.415, PU: β=.165, and AT: β=.163; prefrail group: PEOU: β=.149, PU: β=.276, and AT: β=.201).

However, only the AT score had a significant relationship with the intention to use daily living assistive technologies in the frail group (*r*=0.526; *P*=.002). The fact that the AT score showed significant results, although most factors did not affect the intention to use technology because of the limited number of older adults, confirmed once again that the positive attitude of the older adults had a major influence on the intention to use technology.

## Discussion

### Principal Findings

This study investigated whether there is a relationship between various factors (technological context factors, health contexts and abilities, and attitudinal factors) and behavioral intention to use daily living assistive technologies. The results of the multivariate analyses showed that technological context factors, health contexts and abilities, and attitudinal factors had significant effects on behavioral intention to use daily living assistive technologies. The technological context factors affected behavioral intention to use technology in the robust and prefrail groups, and the health contexts and abilities factors affected behavioral intention to use technology in the prefrail group. Moreover, the attitudinal factors affected all groups (robust, prefrail, and frail).

Our study has revealed that attitudinal factors had the most significant and consistent influence on behavioral intention to use technology among our sample. This was particularly evident in both the robust and prefrail groups, where all 3 attitudinal variables—AT, PU, and PEOU—were significant predictors of behavioral intention to use technology. Technological context seemed to be the second most important factor, with SE playing a role in determining behavioral intention to use technology among individuals in both the robust and prefrail groups. Health factors had relatively small effects on behavioral intention to use technology, with only LS being a predictor for the robust group and HC being a predictor for the prefrail group. Interestingly, almost no demographic factors predicted behavioral intention to use technology in either group. These findings align with previous research emphasizing the critical role of attitudinal factors in predicting older adults’ intention to use technology. In addition, it is noteworthy that, when comparing the 3 groups, no variables other than AT had a significant association with behavioral intention to use technology among older adults in the frail group. This is in contrast to findings among healthier groups, where several other variables contribute to their behavioral intention to use technology.

In addition, we confirmed that there were differences in the intention to use daily living assistive technologies as well as the predictive factors according to the sample’s health status based on their frailty. First, the result that the older adults in the prefrail group had a higher intention to use daily living assistive technologies when their subjective health was poorer was interpreted as an increased intention to use daily living assistive technologies to solve unmet needs. As the older adults in the prefrail group were not as healthy as those in the robust group, they tended to feel that their health was gradually deteriorating. Conversely, no significant relationship was found for the subjective health status of the older adults in the frail group because they already had a lower health status. Therefore, it is necessary to allow older adults to use daily living assistive technologies as a preventive approach according to their characteristics before they enter the frail stage. However, the use rate of daily living assistive technologies in South Korea is quite low because most of the device users are long-term health care insurance beneficiaries. Older adults who use long-term health care insurance can receive information on welfare equipment, and other older adults can buy or rent welfare equipment, but they cannot receive government support for the cost involved; most older adults have no information about what assistive technology products are available, how they can be purchased, and which ones are better. Therefore, a platform that provides product information, education, and product knowledge is also required.

Second, as a result of the significant relationship between SE and behavioral intention to use technology, it can be concluded that the relatively healthier groups had a higher tendency to lead their own lives. It is necessary to inform them well in the early stage of older adult life to use daily living assistive technologies with the help of various types of manuals so that they can use these technologies well with self-initiation and confidence. Recently, in South Korea, many households with middle-aged and older adults received artificial intelligence speakers through an agency that installs internet service, but most of these adults do not use the speakers well because they do not know exactly how to use them. To increase the intention to use this device and to encourage continual use, it is necessary to provide various types of learning methods, such as user manuals written using large letters and simple words as well as audio and video guidance, when purchasing and installing the device.

This study makes several important contributions to the existing literature on technology use among older adults. It is one of the first studies to examine the factors that influence the use of daily living assistive technologies among this population. Our findings highlight that both attitudinal and technological context factors are important determinants of behavioral intention to use such technologies. Furthermore, we observed that both the number and size of significant predictors of behavioral intention to use technology differed according to an individual’s frailty status. These results suggest that it may be more effective to consider the heterogeneity within the older adult population, rather than treating all older adults as a single group, when studying technology use. In particular, in South Korea, only some public health centers and welfare centers are conducting programs related to frailty to prevent and intervene in frailty [38]. In addition, the general public does not recognize the problem and symptoms of frailty [38]. As frailty is a significant indicator of disability or mortality [10,11,29], it is crucial to study frailty by classifying it in the South Korean context. In addition, our findings provide practical guidance for strategies to increase technology use among older adults. Our results indicate that AT, PEOU, and PU are critical factors in determining the intention to use daily living assistive technology products. Therefore, it is important to provide older adults with a range of experiences in various settings, such as welfare centers, department stores, and convenience stores for older adults. This can help to familiarize older adults with newly developed products and promote more positive perceptions of using them. Although South Korea already has several large age-friendly experience centers, it may also be beneficial to manage smaller-scale experience halls to increase older adults’ access to, and experience with, technology.

### Strengths and Limitations

Regarding the strengths of this study, we used a nationwide sample collected from 17 representative cities and provinces in South Korea, and thus we provided a basis for generalizing the results of the study to older South Korean adults through the data. Another strength of this study was that we examined the intention to use daily living assistive technology devices in a multidimensional domain. By examining the dimensions of the STAM, which are technological context factors, health contexts and abilities, and attitudinal factors, we investigated multidimensional aspects of older adults’ behavioral intention to use technology. Finally, this study classified older adults into robust, prefrail, and frail groups, and it examined in detail what factors affected the intention to use technology according to the level of frailty. This study used differentiation to promote the intention to use technology according to the frailty group, and a more detailed approach was provided.

It is important to examine the limitations of this study. This study includes 3 limitations. First, this study identified the technology acceptance factors according to the type of frailty and suggested the implications of accessing daily living assistive technologies by impairment. However, this study includes a limitation in that it did not conduct in-depth interviews by type of frailty. Therefore, in a follow-up study, it is necessary to conduct an in-depth analysis by interviewing older adults by type of frailty. Second, researchers interpret frailty as a combination of problems in different domains of human functioning, such as physical, sensory, psychological, and social domains [39,40]. However, this study used the simple frailty questionnaire measurement method proposed by Morley et al [29]. This scale can easily measure frailty and has been verified by many researchers [41,42]. However, in future studies, it is necessary to use indicators that measure frailty multidimensionally to measure frailty comprehensively. Finally, we could not present the structural equation model result as the main result because the model fit was relatively poor (Multimedia Appendix 4). There needs to be in-depth verification of the relationships among the variables using structural equations in subsequent studies by applying a shortened version of the STAM [43] or South Korean TAM [44] in the follow-up research.

### Conclusions

Our study found a significant relationship between STAM dimension factors and the intention to use daily living assistive technologies among older adults living in communities in South Korea. In particular, this study confirmed that the factors that affected the intention to use were different among the robust, prefrail, and frail groups and provided preliminary evidence of a means of reducing the risk of exacerbating frailty.

## Appendix

Multimedia Appendix 1 Measurement variables, items, and sources.

Multimedia Appendix 2 The overall senior technology acceptance model results.

Multimedia Appendix 3 Results of hierarchical regression analysis of the 3 groups.

Multimedia Appendix 4 Structural equations.

## Abbreviations

ANX: gerontechnology anxiety
AT: attitude toward use
ATT: attitude toward aging
CA: cognitive ability
FC: facilitating conditions
HC: self-reported health conditions
IADL: instrumental activity of daily living
LS: life satisfaction
PEOU: perceived ease of use
PU: perceived usefulness
SE: gerontechnology self-efficacy
STAM: senior technology acceptance model
TAM: technology acceptance model

## Data Availability

The data sets generated or analyzed in this study are available from the corresponding author on reasonable request.
