# Supplementary material for: Comprehensive Senior Technology Acceptance Model of Daily Living Assistive Technology for Older Adults With Frailty: Cross-sectional Study
Source: J Med Internet Res. 2023 Apr 10;25:e41935. doi: 10.2196/41935 (PMC10131916; doi:10.2196/41935)
Supplement: Multimedia Appendix 1 [file jmir_v25i1e41935_app1.docx]

**Multimedia Appendix 1.** Measurement variables, items, and sources.

| Variables | | | | | | | | Cronbach α | Sources |
| --- | --- | --- | --- | --- | --- | --- | --- | --- | --- |
| **BI^a^** | | | | | | | | .9086 | [26] |
|  | | | | | BI 1: I intend to use technology when I need care | | |  |  |
|  | | | | | BI 2: I plan to use technology when I need care | | |  |  |
| **AT^b^** | | | | | | | | .7967 | [21,22] |
|  | | | | | AT 1: using technology is a good idea | | |  |  |
|  | | | | | AT 2: you like the idea of using technology | | |  |  |
| **PU^c^** | | | | | | | | .7967 | [21,22] |
|  | | | | | PU 1: using technology would enhance your effectiveness in life | | |  |  |
|  | | | | | PU 2: using technology would make your life more convenient | | |  |  |
|  | | | | | PU 3: you would find technology useful in your life | | |  |  |
| **PEOU^d^** | | | | | | | | .8725 | [21,22] |
|  | | | | | PEOU 1: you would find that technology is easy to use | | |  |  |
|  | | | | | PEOU 2: you could be skillful at using technology | | |  |  |
| **SE^e^** | | | | | | | | .8919 | [22,26] |
|  | | | | | SE 1: you could complete a task using technology if there is someone to demonstrate how | | |  |  |
|  | | | | | SE 2: you could complete a task using technology if you have just the instruction manual for assistance | | |  |  |
| **ANX^f^** | | | | | | | | .8981 | [22,26] |
|  | | | | | ANX 1: you feel apprehensive about using the technology | | |  |  |
|  | | | | | ANX 2: you hesitate to use the technology for fear of making mistakes you cannot correct | | |  |  |
| **FC^g^** | | | | | | | | .8801 | [22,26] |
|  | | | | | FC 1: you have the knowledge necessary to use the system | | |  |  |
|  | | | | | FC 2: the technology is not compatible with other technologies I use | | |  |  |
|  | | | | | FC 3: a specific person is available for assistance with technology difficulties | | |  |  |
| **HC^h^** | | | | | | | | .7561 | —^i^ |
|  | | HC 1: how is your physical health conditions? | | | | | |  |  |
|  | | HC 2: how is your mental health conditions? | | | | | |  |  |
| **CA^j^** | | | | | | | | .7483 | [31] |
|  | | | CA 1: I have forgotten a story or event I just heard | | | | |  |  |
|  | | | CA 2: I do not know what month it is today | | | | |  |  |
|  | | | CA 3: I do not know where I am | | | | |  |  |
|  | | | CA 4: I do not know my age or birthday | | | | |  |  |
|  | | | CA 5: I do not understand other people’s instructions | | | | |  |  |
|  | | | CA 6: poor judgment about a given situation | | | | |  |  |
|  | | | CA 7: I have a problem with communication | | | | |  |  |
|  | | | CA 8: I cannot count | | | | |  |  |
|  | | | CA 9: I do not understand the daily routine | | | | |  |  |
|  | | | CA 10: I do not recognize family or relatives | | | | |  |  |
| **SR^k^** | | | | | | | | .6920 | [32,33] |
|  | SR 1: in a typical week, how many times do you talk on the telephone with family, friends, or neighbors? | | | | | | |  |  |
|  | SR 2: how often do you get together with friends or relatives? | | | | | | |  |  |
| **Psychological function 1: ATT^l^** | | | | | | | | .7264 | [34] |
|  | | | | | | | ATT 1: as people get older, they are better able to cope with life |  |  |
|  | | | | | | | ATT 2: it is a privilege to grow old |  |  |
|  | | | | | | | ATT 3: old age is a time of loneliness^m^ |  |  |
|  | | | | | | | ATT 4: wisdom comes with age |  |  |
|  | | | | | | | ATT 5: there are many pleasant things about growing older |  |  |
|  | | | | | | | ATT 6: old age is a depressing time of life^m^ |  |  |
|  | | | | | | | ATT 7: it is important to exercise at any age |  |  |
|  | | | | | | | ATT 8: growing older has been easier than I thought |  |  |
|  | | | | | | | ATT 9: I find it more difficult to talk about my feelings as I get older^m^ |  |  |
|  | | | | | | | ATT 10: I am more accepting of myself as I have grown older |  |  |
|  | | | | | | | ATT 11: I do not feel old |  |  |
|  | | | | | | | ATT 12: I see old age mainly as a time of loss^m^ |  |  |
|  | | | | | | | ATT 13: my identity is not defined by my age |  |  |
|  | | | | | | | ATT 14: I have more energy now than I expected for my age |  |  |
|  | | | | | | | ATT 15: I am losing my physical independence as I get older^m^ |  |  |
|  | | | | | | | ATT 16: problems with my physical health do not hold me back from doing what I want |  |  |
|  | | | | | | | ATT 17: as I get older, I find it more difficult to make new friends^m^ |  |  |
|  | | | | | | | ATT 18: it is very important to pass on the benefits of my experiences to younger people |  |  |
|  | | | | | | | ATT 19: I believe my life has made a difference |  |  |
|  | | | | | | | ATT 20: I do not feel involved in society now that I am older^m^ |  |  |
|  | | | | | | | ATT 21: I want to set a good example to younger people |  |  |
|  | | | | | | | ATT 22: I feel excluded from things because of my age^m^ |  |  |
|  | | | | | | | ATT 23: my health is better than I expected for my age |  |  |
|  | | | | | | | ATT 24: I keep as fit and active as possible by exercising |  |  |
| **Psychological function 2: LS^n^** | | | | | | | | .8449 | [35] |
|  | | | | | | LS 1: I lead a purposeful and meaningful life | |  |  |
|  | | | | | | LS 2: my social relationships are supportive and rewarding | |  |  |
|  | | | | | | LS 3: I am engaged and interested in my daily activities | |  |  |
|  | | | | | | LS 4: I actively contribute to the happiness and well-being of others | |  |  |
|  | | | | | | LS 5: I am competent and capable in the activities that are important to me | |  |  |
|  | | | | | | LS 6: I am a good person and live a good life | |  |  |
|  | | | | | | LS 7: my material life (income, housing, etc) is sufficient for my needs | |  |  |
|  | | | | | | LS 8: I generally trust others and feel part of my community | |  |  |
|  | | | | | | LS 9: I am satisfied with my religious or spiritual life | |  |  |
|  | | | | | | LS 10: I am optimistic about the future | |  |  |
|  | | | | | | LS 11: I have no addictions, such as to alcohol, illicit drugs, or gambling | |  |  |
|  | | | | | | LS 12: people respect me | |  |  |
| **Physical function: IADL^o^** | | | | | | | | .8276 | [22,36,37] |
|  | | | | IADL 1: doing housework or handyman work | | | |  |  |
|  | | | | IADL 2: food preparation | | | |  |  |
|  | | | | IADL 3: laundry | | | |  |  |
|  | | | | IADL 4: managing money | | | |  |  |
|  | | | | IADL 5: grocery shopping | | | |  |  |
|  | | | | IADL 6: ability to use the telephone | | | |  |  |
|  | | | | IADL 7: using transportation | | | |  |  |
|  | | | | IADL 8: getting to places beyond walking distance | | | |  |  |
|  | | | | IADL 9: grooming | | | |  |  |
|  | | | | IADL 10: taking medications | | | |  |  |

^a^BI: behavioral intention to use technology.

^b^AT: attitude toward use.

^c^PU: perceived usefulness.

^d^PEOU: perceived ease of use.

^e^SE: gerontechnology self-efficacy.

^f^ANX: gerontechnology anxiety.

^g^FC: facilitating conditions.

^h^HC: self-reported health conditions.

^i^Not available.

^j^CA: cognitive ability.

^k^SR: social relationships.

^l^ATT: attitude toward aging.

^m^Reverse scored.

^n^LS: life satisfaction.

^o^IADL: instrumental activity of daily living.
