# Supplementary material for: Comprehensive Senior Technology Acceptance Model of Daily Living Assistive Technology for Older Adults With Frailty: Cross-sectional Study
Source: J Med Internet Res. 2023 Apr 10;25:e41935. doi: 10.2196/41935 (PMC10131916; doi:10.2196/41935)
Supplement: Multimedia Appendix 2 [file jmir_v25i1e41935_app2.docx]

**Multimedia Appendix 2.** The overall senior technology acceptance model results

**Attitudinal factors**: Perceived usefulness (PU) was the highest-rated positive factor in the attitudinal factor group (Mean = 11.35, 95% CI 11.17–11.53). Attitude toward using (AT) and behavioral intention to use (BI) were the second-highest-rated factors (AT: Mean = 7.438, 95% CI 7.30–7.57; BI: Mean = 7.002, 95% CI 6.84–7.16). However, the perceived ease of use (PEOU) was rated quite low in the attitudinal factor group (Mean = 6.354, 95% CI 6.18–6.52).

**Technological context factors**: Facilitating conditions (Mean = 8.668, 95% CI 8.41–8.92) had a higher score than gerontechnology self-efficacy (Mean = 7.028, 95% CI 6.85–7.21) in the technological context factor group. Gerontechnology anxiety (ANX) was a negative factor in this group, and the mean was 5.162 (95% CI 4.98–5.34).

**Health contexts and abilities**: Self-reported health conditions (HC) had an average of 7.12 (95% CI 7.00–7.25), and cognitive ability (CA) had an average of 19.238 (95% CI 19.12–19.36). Moreover, the mean of social relationships (SR) was 6.678 (95% CI 6.47–6.88, Cronbach’s Alpha = 0.6920). The mean of psychological function 1 (ATT) was 74.256 (95% CI 73.62–74.90), and that of psychological function 2 (LS) was 41.646 (95% CI 41.12–42.18). Finally, IADL had an average of 10.442 (95% CI 10.31–10.58).

Table 1. The overall STAM results (N = 500).

| Factors | Mean | SE | 95% CI |
| --- | --- | --- | --- |
|  |  |  |  |
| **Attitudinal factors** |  |  |  |
| Behavioral intention to use (BI) | 7.002 | 0.081 | 6.84-7.16 |
| Attitude toward use (AT) | 7.438 | 0.068 | 7.30-7.57 |
| Perceived usefulness (PU) | 11.35 | 0.094 | 11.17-11.53 |
| Perceived ease of use (PEOU) | 6.354 | 0.087 | 6.18-6.52 |
| **Technological context factors** |  |  |  |
| Gerontechnology self-efficacy (SE) | 7.028 | 0.091 | 6.85-7.21 |
| Gerontechnology anxiety (ANX) | 5.162 | 0.092 | 4.98-5.34 |
| Facilitating conditions (FC) | 8.668 | 0.132 | 8.41-8.92 |
| **Health contexts and abilities** |  |  |  |
| Self-reported health conditions (HC) | 7.128 | 0.065 | 7.00-7.25 |
| Cognitive ability (CA) | 19.238 | 0.060 | 19.12-19.36 |
| Social relationships (SR) | 6.678 | 0.105 | 6.47-6.88 |
| Psychological function 1 (ATT) | 74.256 | 0.326 | 73.62-74.90 |
| Psychological function 2 (LS) | 41.646 | 0.270 | 41.12-42.18 |
| Physical function (IADL) | 10.442 | 0.068 | 10.31-10.58 |
