# Supplementary material for: Comprehensive Senior Technology Acceptance Model of Daily Living Assistive Technology for Older Adults With Frailty: Cross-sectional Study
Source: J Med Internet Res. 2023 Apr 10;25:e41935. doi: 10.2196/41935 (PMC10131916; doi:10.2196/41935)
Supplement: Multimedia Appendix 3 [file jmir_v25i1e41935_app3.docx]

**Multimedia Appendix 3.** Results of hierarchical regression analysis of the 3 groups.

Table 1. Results of hierarchical regression analysis of the robust group (n = 226).

|  | Factors | Model 1 | | Model 2 | | Model 3 | | Model 4 | |
| --- | --- | --- | --- | --- | --- | --- | --- | --- | --- |
|  |  | Coef.  (SE) | P value | Coef.  (SE) | P value | Coef.  (SE) | P value | Coef.  (SE) | P value |
|  |  |  |  |  |  |  |  |  |  |
| 1 | Age | 0.055  (0.019) | .004 | -0.256  (0.013) | .04 | -0.021  (0.013) | .11 | -.016  (0.013) | .24 |
|  | Gender | 0.012  (0.242) | .86 | 0.201  (0.160) | .21 | 0.229  (0.160) | .15 | 0.241  (0.164) | .14 |
|  | Educational attainment | 0.290  (0.146) | .047 | -0.016  (0.098) | .87 | -0.042  (0.100) | .68 | -0.007  (0.103) | .94 |
|  | Marital status | 0.327  (0.313) | .97 | -0.245  (0.207) | .24 | -0.232  (0.206) | .26 | -0.223  (0.206) | .28 |
|  | Working | -0.699  (0.283) | .014 | -0.301  (0.190) | .11 | -0.261  (0.190) | .17 | -0.233  (0.192) | .22 |
|  | Monthly household income | 0.000  (0.000) | .79 | 0.000  (0.000) | .74 | 0.000  (0.000) | .74 | 0.000  (0.000) | .48 |
| 2 | Attitude toward use (AT) |  |  | 0.219  (0.086) | .011 | 0.216  (0.085) | .012 | 0.186  (0.086) | .03 |
|  | Perceived usefulness (PU) |  |  | 0.372  (0.064) | <.001 | 0.345  (0.066) | <.001 | 0.348  (0.066) | <.001 |
|  | Perceived ease of use (PEOU) |  |  | 0.220  (0.054) | <.001 | 0.159  (0.062) | .012 | 0.162  (0.062) | .01 |
| 3 | Gerontechnology self-efficacy (SE) |  |  |  |  | 0.111  (0.054) | .04 | 0.122  (0.055) | .03 |
|  | Gerontechnology anxiety (ANX) |  |  |  |  | -0.003  (0.045) | .95 | 0.014  (0.046) | .77 |
|  | Facilitating conditions (FC) |  |  |  |  | 0.027  (0.038) | .48 | 0.045  (0.039) | .24 |
| 4 | Self-reported health conditions (HC) |  |  |  |  |  |  | 0.074  (0.067) | .27 |
|  | Cognitive ability (CA) |  |  |  |  |  |  | 0.076  (0.064) | .23 |
|  | Social relationships (SR) |  |  |  |  |  |  | 0.052  (0.033) | .12 |
|  | Psychological function 1 (ATT) |  |  |  |  |  |  | -0.005  (0.013) | .70 |
|  | Psychological function 2 (LS) |  |  |  |  |  |  | -0.040  (0.017) | .02 |
|  | Physical function (IADL) |  |  |  |  |  |  | -0.024  (0.089) | .79 |
|  | (constant) | 10.168  (1.106) | <.001 | 1.594  (1.230) | .20 | 0.964  (1.322) | .47 | 0.254  (2.045) | .90 |
|  | R²(Adj R²) | 0.108(0.083) | | 0.620(0.604) | | 0.630(0.609) | | 0.648(0.617) | |
|  | F | 4.42 | | 39.19 | | 30.20 | | 21.13 | |

Table 2. The Hierarchical regression analysis results of the prefrail group (n = 212).

|  | Factors | Model 1 | | Model 2 | | Model 3 | | Model 4 | |
| --- | --- | --- | --- | --- | --- | --- | --- | --- | --- |
|  |  | Coef.  (SE) | P value | Coef.  (SE) | P value | Coef.  (SE) | P value | Coef.  (SE) | P value |
|  |  |  |  |  |  |  |  |  |  |
| 1 | Age | -0.005  (0.019) | .78 | 0.014  (0.013) | .28 | 0.024  (0.012) | .057 | 0.025  (0.013) | .05 |
|  | Gender | -0.065  (0.255) | .80 | 0.114  (0.174) | .51 | 0.131  (0.163) | .42 | 0.101  (0.170) | .55 |
|  | Educational attainment | 0.239  (0.145) | .10 | 0.138  (0.099) | .16 | 0.112  (0.095) | .24 | 0.132  (0.098) | .18 |
|  | Marital status | -0.128  (0.317) | .69 | -0.609)  (0.218) | .006 | -0.691  (0.205) | .001 | -0.715  (0.209) | .001 |
|  | Working | -0.280  (0.306) | .36 | -0.058  (0.216) | .79 | 0.032  (0.203) | .88 | 0.065  (0.209) | .75 |
|  | Monthly household income | 0.000  (0.000) | .81 | 0.000  (0.000) | .75 | 0.000  (0.000) | .42 | 0.000  (0.000) | .18 |
| 2 | Attitude toward use (AT) |  |  | 0.316  (0.087) | <.001 | 0.252  (0.083) | .003 | 0.235  (0.084) | .006 |
|  | Perceived usefulness (PU) |  |  | 0.149  (0.059) | .013 | 0.106  (0.056) | .06 | 0.120  (0.057) | .04 |
|  | Perceived ease of use (PEOU) |  |  | 0.444  (0.057) | <.001 | 0.302  (0.063) | <.001 | 0.265  (0.065) | <.001 |
| 3 | Gerontechnology self-efficacy (SE) |  |  |  |  | 0.307  (0.058) | <.001 | 0.331  (0.059) | <.001 |
|  | Gerontechnology anxiety (ANX) |  |  |  |  | -0.029  (0.049) | .56 | -0.028  (0.051) | .58 |
|  | Facilitating conditions (FC) |  |  |  |  | -0.025  (0.045) | .58 | -0.021  (0.045) | .64 |
| 4 | Self-reported health conditions (HC) |  |  |  |  |  |  | -0.169  (0.068) | .013 |
|  | Cognitive ability (CA) |  |  |  |  |  |  | 0.107  (0.083) | .20 |
|  | Social relationships (SR) |  |  |  |  |  |  | -0.020  (0.037) | .59 |
|  | Psychological function 1 (ATT) |  |  |  |  |  |  | 0.017  (0.014) | .22 |
|  | Psychological function 2 (LS) |  |  |  |  |  |  | -0.003  (0.015) | .86 |
|  | Physical function (IADL) |  |  |  |  |  |  | -0.085  (0.084) | .31 |
|  | (constant) | 6.929  (1.733) | <0.001 | -0.973  (1.293) | .47 | -1.527  (1.238) | .22 | -2.684  (2.248) | .23 |
|  | R²(Adj R²) | 0.021(-0.007) | | 0.556(0.537) | | 0.618(0.595) | | 0.637(0.603)) | |
|  | F | 0.74 | | 28.15 | | 26.88 | | 18.79 | |

Table 3. The Hierarchical regression analysis results of the frail group (n = 62).

|  | Factors | Model 1 | | Model 2 | | Model 3 | | Model 4 | |
| --- | --- | --- | --- | --- | --- | --- | --- | --- | --- |
|  |  | Coef.  (SE) | P value | Coef.  (SE) | P value | Coef.  (SE) | P value | Coef.  (SE) | P value |
|  |  |  |  |  |  |  |  |  |  |
| 1 | Age | -0.075  (0.042) | .08 | -0.003  (0.303) | .93 | -0.011  (0.030) | .72 | -0.007  (0.035) | .85 |
|  | Gender | -0.094  (0.588) | .87 | -0.264  (0.412) | .52 | -0.271  (0.423) | .52 | -0.165  (0.485) | .73 |
|  | Educational attainment | 0.080  (0.342) | .82 | 0.138  (0.261) | .60 | 0.092  (0.265) | .73 | 0.129  (0.305) | .67 |
|  | Marital status | 0.092  (0.579) | .87 | -0.100  (0.403) | .80 | -0.083  (0.400) | .84 | -0.074  (0.440) | .87 |
|  | Working | -0.101  (0.690) | .88 | 0.346  (0.488) | .78 | 0.307  (0.504) | .55 | 0.421  (0.568) | .46 |
|  | Monthly household income | -0.005  (0.002) | .03 | -0.002  (0.001) | .11 | -0.002  (0.001) | .09 | -0.002  (0.002) | .17 |
| 2 | Attitude toward use (AT) |  |  | 0.474  (0.138) | .001 | 0.512  (0.137) | .001 | 0.526  (0.163) | .002 |
|  | Perceived usefulness (PU) |  |  | 0.225  (0.110) | .04 | 0.207  (0.111) | .07 | 0.170  (0.135) | .22 |
|  | Perceived ease of use (PEOU) |  |  | 0.381  (0.105) | .001 | 0.090  (0.174) | .61 | 0.085  (0.221) | .70 |
| 3 | Gerontechnology self-efficacy (SE) |  |  |  |  | 0.057  (0.126) | .65 | 0.068  (0.148) | .65 |
|  | Gerontechnology anxiety (ANX) |  |  |  |  | -0.081  (0.114) | .48 | -0.059  (0.129) | .65 |
|  | Facilitating conditions (FC) |  |  |  |  | 0.193  (0.116) | .10 | 0.191  (0.127) | .14 |
| 4 | Self-reported health conditions (HC) |  |  |  |  |  |  | -0.044  (0.176) | .80 |
|  | Cognitive ability (CA) |  |  |  |  |  |  | 0.034  (0.107) | .75 |
|  | Social relationships (SR) |  |  |  |  |  |  | 0.031  (0.096) | .75 |
|  | Psychological function 1 (ATT) |  |  |  |  |  |  | 0.007  (0.036) | .84 |
|  | Psychological function 2 (LS) |  |  |  |  |  |  | 0.007  (0.050) | .89 |
|  | Physical function (IADL) |  |  |  |  |  |  | 0.058  (0.089) | .52 |
|  | (constant) | 12.603  (4.080) | 0.003 | -0.797  (3.42) | .72 | 0.283  (3.571) | .94 | -2.218  (6.249) | .72 |
|  | R²(Adj R²) | 0.134(0.040) | | 0.616(0.550) | | 0.650(0.564) | | 0.657(0.513) | |
|  | F | 1.42 | | 9.27 | | 7.58 | | 4.57 | |
