# Supplementary material for: Comprehensive Senior Technology Acceptance Model of Daily Living Assistive Technology for Older Adults With Frailty: Cross-sectional Study
Source: J Med Internet Res. 2023 Apr 10;25:e41935. doi: 10.2196/41935 (PMC10131916; doi:10.2196/41935)
Supplement: Multimedia Appendix 4 [file jmir_v25i1e41935_app4.docx]

**Multimedia Appendix 4.** Structural equations.

Table 1. The factor load, Cronbach’s alpha value of each variable.

| Variables | Factor load | Cronbach's alpha |
| --- | --- | --- |
|  |  |  |
| **Behavioral intention to use (BI)** |  | 0.9086 |
| BI 1. I intend to use technology when I need care | 0.9581 |  |
| BI 2. I plan to use technology when I need care | 0.9581 |  |
| **Attitude toward using (AT)** |  | 0.7967 |
| AT 1. Using technology is a good idea | 0.9132 |  |
| AT 2. You like the idea of using technology | 0.9132 |  |
| **Perceived usefulness (PU)** |  | 0.7967 |
| PU1. Using technology would enhance your effectiveness in life | 0.8777 |  |
| PU2. Using technology would make your life more convenient | 0.8977 |  |
| PU3. You would find technology useful in your life | 0.9042 |  |
| **Perceived ease of use (PEOU)** |  | 0.8725 |
| PEOU 1. You would find technology is easy to use | 0.9506 |  |
| PEOU 2. You could be skillful at using technology | 0.9506 |  |
| **Gerontechnology self-efficacy (SE)** |  | 0.8919 |
| SE 1. You could complete a task using technology if there is someone to demonstrate how | 0.9533 |  |
| SE 2. You could complete a task using technology if you have just the instruction manual for assistance | 0.9533 |  |
| **Gerontechnology anxiety (ANX)** |  | 0.8981 |
| ANX 1. You feel apprehensive about using the technology | 0.9452 |  |
| ANX 2. You hesitate to use the technology for fear of making mistakes you cannot correct | 0.9452 |  |
| **Facilitating conditions (FC)** |  | 0.8801 |
| FC 1. You have the knowledge necessary to use the system | 0.8915 |  |
| FC 2. The technology is not compatible with other technology I use. | 0.8023 |  |
| FC 3. A specific person is available for assistance with technology difficulties | 0.8522 |  |
| **Health Conditions (HC)** |  | 0.7561 |
| HC 1. How are your physical health conditions? | 0.8976 |  |
| HC 2. How are your mental health conditions? | 0.8976 |  |
| **Cognitive ability (CA)** |  | 0.7483 |
| CA 1. I have forgotten a story or event I just heard | 0.3869 |  |
| CA 2. I don't know what month it is today | 0.6332 |  |
| CA 3. I don't know where I am | 0.7402 |  |
| CA 4. I don't know my age or birthday | 0.7268 |  |
| CA 5. I do not understand other people's instructions | 0.691 |  |
| CA 6. Poor judgment about a given situation | 0.5232 |  |
| CA 7. I have a problem with communication | 0.6463 |  |
| CA 8. I cannot count | 0.5782 |  |
| CA 9. I do not understand the daily routine | 0.752 |  |
| CA 10. I do not recognize family or relatives | 0.6673 |  |
| **Social relationships (SR)** |  | 0.6920 |
| SR 1. In a typical week, how many times do you talk on the telephone with family, friends, or neighbors? | 0.8749 |  |
| SR 2. How often do you get together with friends or relatives? | 0.8749 |  |
| **Psychological function 1. Attitude to aging (ATT)** |  | 0.7264 |
| ATT 1. As people get older they are better able to cope with life | 0.4908 |  |
| ATT 2. It is a privilege to grow old | 0.3529 |  |
| ATT 3. Old age is a time of loneliness ^a^ | 0.4841 |  |
| ATT 4. Wisdom comes with age | 0.4101 |  |
| ATT 5. There are many pleasant things about growing older | 0.4271 |  |
| ATT 6. Old age is a depressing time of life ^a^ | 0.3508 |  |
| ATT 7. It is important to take exercise at any age | 0.4666 |  |
| ATT 8. Growing older has been easier than I thought | 0.3682 |  |
| ATT 9. I find it more difficult to talk about my feelings as I get older ^a^ | 0.5089 |  |
| ATT 10. I am more accepting of myself as I have grown older | 0.6913 |  |
| ATT 11. I don’t feel old | 0.4831 |  |
| ATT 12. I see old age mainly as a time of loss ^a^ | 0.5105 |  |
| ATT 13. My identity is not defined by my age | 0.4497 |  |
| ATT 14. I have more energy now than I expected for my age | 0.5269 |  |
| ATT 15. I am losing my physical independence as I get older ^a^ | 0.5511 |  |
| ATT 16. Problems with my physical health do not hold me back from doing what I want | 0.5002 |  |
| ATT 17. As I get older I find it more difficult to make new friends ^a^ | 0.5348 |  |
| ATT 18. It is very important to pass on the benefits of my experiences to younger people | 0.5738 |  |
| ATT 19. I believe my life has made a difference | 0.4011 |  |
| ATT 20. I don’t feel involved in society now that I am older ^a^ | 0.4823 |  |
| ATT 21. I want to give a good example to younger people | 0.6098 |  |
| ATT 22. I feel excluded from things because of my age ^a^ | 0.3804 |  |
| ATT 23. My health is better than I expected for my age | 0.4859 |  |
| ATT 24. I keep as fit and active as possible by exercising | 0.4816 |  |
| **Psychological function 2. Life satisfaction (LS)** |  | 0.8449 |
| LS 1. I lead a purposeful and meaningful life | 0.7229 |  |
| LS 2. My social relationships are supportive and rewarding | 0.6962 |  |
| LS 3. I am engaged and interested in my daily activities | 0.7026 |  |
| LS 4. I actively contribute to the happiness and well-being of others | 0.6998 |  |
| LS 5. I am competent and capable in the activities that are important to me | 0.7608 |  |
| LS 6. I am a good person and live a good life | 0.6274 |  |
| LS 7. My material life (income, housing, etc.) is sufficient for my needs | 0.6429 |  |
| LS 8. I generally trust others and feel part of my community | 0.6199 |  |
| LS 9. I am satisfied with my religious or spiritual life | 0.4199 |  |
| LS 10. I am optimistic about the future | 0.6984 |  |
| LS 11. I have no addictions, such as to alcohol, illicit drugs, or gambling | 0.1319 |  |
| LS 12. People respect me | 0.6175 |  |
| **Physical function (IADL)** |  | 0.8276 |
| IADL 1. Doing housework or handyman work | 0.7117 |  |
| IADL 2. Food preparation | 0.6701 |  |
| IADL 3. Laundry | 0.636 |  |
| IADL 4. Managing money | 0.667 |  |
| IADL 5. Grocery shopping | 0.7366 |  |
| IADL 6. Ability to use telephone | 0.3452 |  |
| IADL 7. Using transportation | 0.7661 |  |
| IADL 8. Getting to places beyond walking distance | 0.7587 |  |
| IADL 9. Grooming | 0.6972 |  |
| IADL 10. Taking medications | 0.6972 |  |

Table 2. Discriminant validity assessment.^ab^

|  | BI | AT | PU | PEOU | SE | ANX | FC | HC | SR |
| --- | --- | --- | --- | --- | --- | --- | --- | --- | --- |
| BI | 0.836 |  |  |  |  |  |  |  |  |
| AT | 0.563 | 0.669 |  |  |  |  |  |  |  |
| PU | 0.527 | 0.802 | 0.698 |  |  |  |  |  |  |
| PEOU | 0.465 | 0.303 | 0.298 | 0.808 |  |  |  |  |  |
| SE | 0.439 | 0.287 | 0.269 | 0.55 | 0.818 |  |  |  |  |
| ANX | 0.157 | 0.073 | 0.053 | 0.346 | 0.315 | 0.788 |  |  |  |
| FC | 0.41 | 0.318 | 0.297 | 0.683 | 0.579 | 0.415 | 0.594 |  |  |
| HC | 0.015 | 0.018 | 0.007 | 0.101 | 0.104 | 0.208 | 0.155 | 0.640 |  |
| SR | 0.064 | 0.055 | 0.038 | 0.053 | 0.052 | 0.08 | 0.078 | 0.001 | 0.605 |

^a^The AVE(Average variance extraction) values are shown on the diagonals, non-diagonal elements are the SC(Squared correlations).

^b^Excluding variables that are difficult to use in the model due to their low factor loading values.

Table 3. Path test of the structural equation.

| Path | | | Path Coefficient | S.E | P value |
| --- | --- | --- | --- | --- | --- |
|  |  |  |  |  |  |
| AT | → | BI | 0.470 | 0.163 | .004 |
| PU | → | BI | 0.214 | 0.168 | .20 |
| PEOU | → | BI | 0.242 | 0.071 | .001 |
| SE | → | BI | 0.190 | 0.052 | <.001 |
| ANX | → | BI | -0.039 | 0.046 | .40 |
| FC | → | BI | -0.013 | 0.079 | .87 |
| HC | → | BI | -0.109 | 0.043 | .01 |
| SR | → | BI | 0.018 | 0.024 | .45 |
| PU | → | AT | 0.906 | 0.062 | <.001 |
| PEOU | → | AT | 0.002 | 0.060 | .98 |
| SE | → | AT | 0.031 | 0.044 | .48 |
| ANX | → | AT | 0.011 | 0.039 | .77 |
| FC | → | AT | 0.042 | 0.067 | .54 |
| HC | → | AT | 0.027 | 0.036 | .46 |
| SR | → | AT | 0.026 | 0.020 | .20 |
| PEOU | → | PU | 0.180 | 0.068 | .008 |
| SE | → | PU | 0.137 | 0.049 | .005 |
| ANX | → | PU | 0.150 | 0.042 | <.001 |
| FC | → | PU | 0.211 | 0.076 | .005 |
| HC | → | PU | -0.074 | 0.041 | .07 |
| SR | → | PU | 0.029 | 0.025 | .24 |
| SE | → | PEOU | 0.236 | 0.056 | <.001 |
| ANX | → | PEOU | -0.075 | 0.048 | .12 |
| FC | → | PEOU | 0.556 | 0.071 | 0 |
| HC | → | PEOU | -0.041 | 0.045 | .36 |
| SR | → | PEOU | -0.012 | 0.025 | .64 |

| 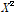=293.827***, RMSEA=0.049, SRMR=0.028, TLI=0.975, CFI=0.965 |
| --- |
